# Severe acute organophosphate poisoning managed with 2-month prolonged atropine therapy: a case report

**DOI:** 10.1097/MS9.0000000000001207

**Published:** 2023-08-16

**Authors:** Bibhav Bashyal, Alisha Yadav, Anand K. Deo, Kirti K. Kharel, Deepshikha Kharel, Bishal Panthi

**Affiliations:** a Tribhuvan University Teaching Hospital, Institute of Medicine, Kathmandu; b Maharajgunj Medical Campus, Institute of Medicine, Maharajgunj; c Nobel Medical College Teaching Hospital; d Manipal College of Medical Sciences, Biratnagar, Nepal

## Abstract

**Introduction and Importance:**

Organophosphate (OP) poisoning is a common and potentially fatal condition that requires prompt and aggressive treatment with atropine, oximes, and supportive care. We report a rare case of OP poisoning that needed high doses of atropine and intensive care for 60 days.

**Case Presentation:**

A 39-year-old male ingested 200 ml of chlorpyrifos, an OP compound, and presented with vomiting and epigastric pain. He received an initial dose of atropine of 60 ml (36 mg, 1 ml=0.6 mg), followed by an infusion of 16 ml/h (9.6 mg/h). He developed hypoxia, cardiac arrest, delirium, fever, and persistent bronchorrhea. He was intubated, resuscitated, and transferred to ICU, where he continued showing signs of OP excess and therefore, he received up to 170 ml/h (102 mg/h) of atropine infusion, along with triple inotropes and sedation. He underwent tracheostomy and gradual weaning of atropine. He recovered completely and was discharged in stable condition.

**Clinical Discussion:**

This case demonstrates the need for prolonged monitoring of patients with OP poisoning wherein the patient can develop signs of OP excess even after initial atropinization, the effectiveness of multiple doses of atropine in OP poisoning, and the importance of monitoring for complications associated with a prolonged hospital stay. It also shows the potential need for prolonged atropine therapy and intensive care in OP poisoning.

**Conclusion:**

OP poisoning can be life-threatening and requires early and aggressive treatment with atropine, oximes, and supportive care. Clinicians should be aware of the potential need for prolonged atropine therapy in OP poisoning cases to improve the chances of survival.

## Introduction

Highlights

- The case highlights the need for early and aggressive treatment with atropine, oximes, and supportive care in organophosphate (OP) poisoning.
- The case demonstrates the possible reemergence of signs of OP excess even after initial atropinization, the effectiveness of multiple doses of atropine in OP poisoning, and the importance of monitoring for complications.
- The patient was successfully weaned off atropine and discharged in stable condition.

Organophosphate (OP) poison is a common and cheap agent for suicide. It poses a major health problem globally and is the most common modality of suicide in Nepal. OP poisoning results from exposure to occupational or accidental sources, deliberate ingestion, or chemical warfare agents. The clinical manifestations differ greatly based on the dose, toxicity, and type of exposure of the OP^1^. OP acts by phosphorylating serine hydroxyl group on acetylcholinesterase enzyme, thus resulting in its inactivation, which causes accumulation of acetylcholine followed by overstimulation of nicotinic and muscarinic receptors^2^. Acute toxidrome in OP poisoning can be characterized by muscarinic symptoms: salivation, lacrimation, urination, defecation, gastric cramps, and emesis, which are collectively known as SLUDGE symptoms or nicotinic symptoms: muscle cramps, twitching, fasciculations, tachycardia, hypertension, and weakness. The chronic complications of OP poisoning are the intermediate syndrome and the OP-induced delayed polyneuropathy^3^.

The treatment of choice for acute OP poisoning is atropine, pralidoxime (2-PAM), and supportive care. OP pesticide poisoning usually needs much higher doses of atropine than other conditions. A fast way to reach the right level of atropine is to double the dose each time, starting from 1 mg and going up to 2, 4, 8, 16 mg, and so on, till atropinization is achieved. 2-PAM is given as 2 g intravenous (i.v.) over 5–10 min followed by 8–10 mg/kg/h i.v. infusion for 48–72 h. Oxygen should be given before atropine to reduce the risk for dysrhythmias^4^.

We report a case of a 39-year-old man who survived acute severe OP poisoning with 2-month atropine therapy, along with pralidoxime and benzodiazepine. This case demonstrates the possible reemergence of signs of OP excess even after initial atropinization and that prolonged atropine therapy can be effective even when pralidoxime and benzodiazepine are also given.

## Case presentation

A 39-year gentleman presented to our center with a chief complaint of vomiting and epigastric pain for 30 min. He has an alleged history of ingestion of OP (chlorpyrifos) 200 ml as a suicidal attempt 1 h prior to the evaluation. Following ingestion of OP, the patient also ingested detergent water solution to self-induce vomiting. He did not give a history of any prior comorbidities. On admission, his general condition was fair [Glasgow Coma Scale (GCS) – E4V5M6 (eye opening – E, verbal response – V, motor response – M)]. Bilateral pupils were 2 mm in diameter, round, regular, and reactive to light. He was alert, conscious, cooperative, and oriented to time, place, and person. His vital signs were stable and within normal range. There were crackles over the bilateral lung field. His heart sounds S1/S2 was normal with no added murmur. His higher mental functions, sensations, cranial nerves, and coordination were normal. The tone, power, and reflexes of his upper and lower limbs were normal. The rest of the systemic examination findings were regular. Investigations such as cholinesterase level, coagulation assay, renal function tests, liver function tests, hematology were done as shown in Table 1

**Table 1:** Investigations.

Tests	Units	Results	Reference range
Cholinesterase	kU/l	0.13	4.6–11.5
PT	s	17	10–12
PT control	s	12	10–12
PT INR		1.25	
Sodium	mEq/l	137	135–146
Potassium	mEq/l	3.6	3.5–5.2
Urea	mmol/l	4.3	2.8–7.2
Creatinine	mmol/l	60	59–104
TLC	/mm^3^	11 700	4000–11 000
Neutrophils	%	78	45–75
Lymphocytes	%	12	25–45
Hb	g%	9.4	12–18
PCV	%	26.5	36–54
RBC	million/mm^3^	2.91	4.5–5.5
Platelets	/mm^3^	496 000	150 000–400 000
Total bilirubin		35	5–21
Direct bilirubin		19	<4
SGPT/ALT	U/l	50	0–50
SGOT/AST	U/l	39	0–50
Alkaline phosphatase	U/l	167	30–120
Total protein	g/l	76	66–83
Albumin	g/l	31	35–52

Hb, hemoglobin; INR, international normalized ratio; PCV, packed cell volume; PT, prothrombin time; RBC, red blood cell; SGOT/AST, serum glutamic oxaloacetic transaminase/aspartate aminotransferase; SGPT/ALT, serum glutamic pyruvic transaminase/alanine aminotransferase; TLC, total leukocyte count.

His condition was identified as OP poisoning. At presentation, the patient received an escalated dose of atropine according to protocol, with the dose doubling every 5 min until atropinization was observed (2 ml–4 ml–8 ml–16 ml–30 ml). The patient showed signs of atropinization after receiving a cumulative dose of 60 ml (36 mg, 1 ml=0.6 mg) of atropine and was then maintained on an atropine infusion of 16 ml/h (9.6 mg/h). The patient was given 2 g of pralidoxime over 10 min followed by continuous infusion at 500 mg/h i.v. infusion. Labs showed the patient's serum acetylcholinesterase level 167 U/l (normal values: 4620–11 500 U/l). The patient was planned for ICU admission for further treatment and monitoring. However, due to the unavailability of an ICU bed at that time, the patient was transferred to another hospital for ICU admission.

As per documents from another hospital and case discussion with the treating physician from another hospital, the maintenance dose could not be reduced for this patient due to persistent symptoms of OP excess. 2-PAM infusion was continued for a total of 72 h. Five days after the first atropinization, the patient developed symptoms of OP excess: bronchorrhea, moist axilla and tongue, miosis, and was atropinized again. He received a cumulative 100 ml (60 mg) of atropine in a sequential escalation fashion and was started on an infusion at 20 ml/h (12 mg/h). An alternative diagnosis of hospital-acquired pneumonia was ruled out as his blood counts were normal, serum procalcitonin was 0.25 ng/ml (normal range: 0–0.5 ng/ml), and his sputum cultures were negative. Two days later, the patient again developed excessive bronchial secretions, moist axilla and tongue, had respiratory distress, and was intubated for airway protection and respiratory support. The patient was reatropinized with a cumulative dose of 385 ml (231 mg) and started on a 77 ml/h (46.2 mg/h) infusion on that day after intubation.

On the ninth day of hospitalization, the patient developed asystole. Cardiopulmonary resuscitation (CPR) was started immediately. After 5 min of CPR, electrocardiogram (ECG) showed ventricular fibrillation (shockable rhythm) and the patient was given a DC (direct current) shock of 150 J. CPR was resumed immediately. On the next check for rhythm, the monitor showed sinus rhythm, and CPR was stopped. Post CPR patient was in a state of shock and therefore inotropes were started. To achieve the target mean arterial pressure of >65 mm of Hg, inotropes were escalated and eventually patient required triple inotropes. As shock gradually improved, inotropes were tapered and by the 12th day of hospitalization patient was maintained at single inotrope infusion: dopamine 10 μg/kg/min. Due to economic issues with continuing treatment, the patient was shifted back to our hospital after 12 days of hospitalization at another center and admitted directly to the ICU of our hospital this time. Upon receiving the patient at ICU, he was intubated, his GCS was E3VTM4, and he was under atropine infusion 100 ml/h (60 mg/h) and dopamine infusion 10 μg/kg/min.

The patient was managed with continuous atropine infusion along with a plan of daily reduction in infusion dose by 10%. But the patient's serum acetylcholinesterase level was persistently low (137 U/l) and the patient would develop signs of OP excess – bibasilar crackles, moist tongue and axilla, excessive secretions, and increased oxygen requirement. This had made daily tapering of atropine really difficult. His atropine infusion had to be increased to a maximum of 170 ml/h (102 mg/h). The patient had prolonged atropine requirement, most likely due to redistribution of OP. It may also be likely due to the fact that he presented early, and only a fraction of OP that had been absorbed was atropinized while the process of absorption of further OP was ongoing, thereby requiring less atropinization dose early on but a larger maintenance dose later. The patient also developed delirium, which was managed with benzodiazepine infusion. In view of the prolonged need for ventilatory support, the patient was tracheostomized after 14 days of intubation. The atropine infusion was reduced by 10% every day, with some dose adjustments as required. After 60 days, the patient was finally off atropine. Administration of multiple doses of atropine, in this case, helped the patient to recover. The patient was discharged in a stable state. He is doing well during follow-up.

## Discussion

OP poisoning is common but rarely fatal and has a good outlook. Early treatment can boost the patient’s chances of survival^5^. OP poisoning can cause different symptoms depending on the receptor affected, the duration of exposure, or the organ system involved^3^. Most patients had muscarinic symptoms, such as excessive salivation and sweating, followed by central nervous system symptoms, such as confusion and seizures, and nicotinic symptoms, such as muscle weakness and twitching^6^. In this case, the patient had vomiting and epigastric pain for 30 min at the time of presentation.

The preferred treatment for OP poisoning is atropine which acts by inhibiting acetylcholine at its muscarinic receptors. 2 Bolus loading dose of 0.6–3 mg atropine is given via i.v. route followed by doubling doses every 5 min until the patient is atropinized [HR (heart rate) >80 bpm, SBP (systolic blood pressure) >80 mmHg, clear lungs on auscultation, dry axilla and tongue]. Atropinization is maintained with an infusion of 10–20% of the total dose needed per hour in saline. For mitigating the nicotinic effect of OP, acetylcholinesterase is required. This is where 2-PAM is necessary. 2-PAM works by restoring the function of the acetylcholinesterase enzyme (AChE). OPs block AChE, so 2-PAM reverses this effect and reduces the nicotinic symptoms, such as muscle spasms and weakness^7^. The patient is monitored for cholinergic or atropine toxicity. If cholinergic activity reoccurs, then the bolus dose is resumed until atropinization is achieved again, and the infusion rate is increased by 20% per hour. If the patient develops atropine toxicity (tachycardia, absent bowel sounds, hyperthermia, delirium, urinary retention), then the infusion is stopped for 30 min and then again restarted at a 20% lower dose^8^.

Here, in this case, the patient visited us after 1 h of ingestion of OP compounds. Initially, he was atropinized with 60 ml (36 mg) of atropine, followed by an infusion of 16 ml/h (9.6 mg/h). There were persistent symptoms of OP excess, so he was reatropinized twice, once 5 days after the first atropinization with 100 ml (60 mg) of atropine and infusion at 20 ml/h (12 mg/h). After 2 days of the second atropinization, he was again reatropinized with 385 ml (231 mg) of atropine and infusion at 77 ml/h (46.2 mg/h).

This case report presents a rare and severe case of OP poisoning that required high doses of atropine and intensive care for 60 days. The patient survived despite developing hypoxia, cardiac arrest, atropine psychoses, and thick secretions. He was successfully weaned off atropine and discharged in stable condition. This case highlights the need for early and aggressive treatment with atropine, the possible reemergence of signs of OP excess even after initial atropinization and the need for prolonged atropine infusion, supportive care in OP poisoning. It also shows the potential complications of atropine therapy and the need for close monitoring and dose adjustment. This case adds to the existing literature on the management of OP poisoning and provides valuable insights for clinicians.

## Conclusion

OP poisoning can be life-threatening and requires early and aggressive treatment with atropine, 2-PAM, and intensive care. This case report shows that a patient with OP poisoning survived after receiving high doses of atropine and intensive care for 60 days. Clinicians should be vigilant about the potential need for atropine therapy for prolonged duration along with oximes and intensive care in OP poisoning cases to improve the chances of survival.

## Ethical approval

This is a case report. Therefore, it did not require ethical approval from the ethics committee.

## Consent

Written informed consent was obtained from the patient for the publication of this case report. A copy of the written consent is available for review by the editor-in-chief of this journal on request.

## Sources of funding

The study did not receive any grant from funding agencies in the public, commercial, or not-for-profit sectors.

## Author contribution

B.B., A.Y., A.K.D., K.K.K., D.K., and B.P.: collected all the required information, reports, and figures; reviewed the literature and contributed to writing and editing the manuscript. All authors read and approved the final manuscript.

## Conflicts of interest disclosure

The authors declare that they have no conflicts of interest.

## Research registration unique identifying number (UIN)

1. Name of the registry: not applicable.
2. Unique identifying number or registration ID: not applicable.
3. Hyperlink to your specific registration (must be publicly accessible and will be checked): not applicable.

## Guarantor

Alisha Yadav.

## Provenance and peer review

Not commissioned, externally peer-reviewed.

## Data availability statement

Not applicable.
